# Fixation for calcaneal tuberosity fracture (beak fracture) using preformed “L-shape” hook plate

**DOI:** 10.3389/fsurg.2026.1745665

**Published:** 2026-03-05

**Authors:** Qiang Zhang, Wei Huang, Zongde Wu

**Affiliations:** 1 Department of Foot and Ankle, Sichuan Provincial Orthopedics Hospital, Chengdu, Sichuan, China; 2 Science and Technology Innovation Center, Shanghai Municipal Hospital of Traditional Chinese Medicine, Shanghai University of Traditional Chinese Medicine, Shanghai, China

**Keywords:** avulsion, beak fracture, calcaneus fracture, hook plate, internal fixation

## Abstract

**Objectives:**

Surgical management of calcaneal tuberosity fractures is challenging, as patient outcomes can be compromised by skin flap necrosis and implant failure. We propose a technique utilizing a prefabricated L-shaped hook plate, which represents an innovative clinical orthopedic surgical approach.

**Methods:**

In this retrospective study, patients with Beavis type II calcaneal tuberosity fractures underwent internal fixation using a preformed “L-shape” hook plate (2015–2020). Data on operative time, complications, and healing time were recorded. Functional outcomes were evaluated using the Ankle Society Ankle-Hindfoot (AOFAS-AH) and Visual Analog Scale (VAS) pain scores.

**Results:**

This study included 15 patients (6 females/9 males; mean age 52.9 ± 11.2 years) with calcaneal tuberosity fractures who underwent internal fixation with a preformed “L-shape” hook plate. At a mean follow-up of 17.1 ± 6.0 months, no postoperative complications—including wound issues, infection, nerve injury, or fixation failure—were observed in any patient. All 15 cases achieved clinical healing at an average of 10.5 weeks (range: 8–13). Functional outcomes improved significantly, with the AOFAS-AH score increasing from 24.0 ± 9.9 preoperatively to 93.8 ± 5.2 postoperatively, and the VAS score decreasing from 5.7 ± 0.6 to 1.3 ± 0.5 (*p* < 0.001 for both).

**Conclusions:**

Emergency open reduction and internal fixation is recommended for calcaneal avulsion fractures to prevent flap necrosis. For Beavis type II fractures, the preformed L-shaped hook plate represents a novel and promising alternative, demonstrating favorable early clinical outcomes in this initial series.

## Introduction

Calcaneal avulsion fracture involves bony insertion of Achilles tendon, which does not extend into subtalar joint (1). Calcaneal tuberosity fractures exhibit a higher incidence among elderly female patients, which is typically associated with conditions of impaired bone quality, such as osteoporosis, neuropathy, and/or diabetes (2–4). The main cause of calcaneal tuberosity fracture which is most commonly seen is forced ankle dorsiflexion (4). Calcaneal tuberosity displacement usually induces local excessive tension and/or soft tissue irritation, which may further result in local flap necrosis (5, 6). Although calcaneal tuberosity fractures with minimal displacement may not require surgical treatment, more severe displacement necessitates immediate open reduction and internal fixation (ORIF) to alleviate soft tissue tension, avoid iatrogenic skin injury, and ensure the integrity of the gastrocnemius-soleus complex (4, 7). Complications such as skin flap necrosis and implant failure not only increase the risk of nonunion or malunion of the calcaneal tuberosity fracture, impairing early ankle mobilization, but can also progress to severe tissue infection and, in the worst cases, necessitate amputation (5, 8). Early and in-time surgical treatment is not only conductive to reducing the incidence of soft tissue necrosis, but helps restoring the normal function of triceps surae, thereby facilitating the rehabilitation of patients’ ankle functions (9, 10). Regarding the treatment of avulsion fractures of calcaneal tuberosity, there is no “one-size-fits-all” therapy in the existed techniques that are still evolving. Operative repair of displaced avulsed fragments usually involves open reduction and internal fixation in order to maintain Achilles tendon functional length and to minimize loss of reduction.

The most common and conventional operative technique treating avulsion fractures of calcaneal tuberosity (beak fracture) is adopting lag screw fixation followed with non-weight-bearing in short-leg cast (9, 11). Unfortunately, the above approach often meets with limited success due to certain problems such as poor bone stock, lack of available bone for screw fixation, etc. With respect to traumatic injuries, loss of reduction is a major concern that should pay particular attention to. Alternative fixation techniques use Ilizarov external fixation and cerclage wire, tension band technique (12, 13). However, performing those techniques is often accompanied with comparatively large incisions, which may further increase the trauma and induce severe skin complications.

In response to the abovementioned problems, in this study, we propose an improved approach for treating avulsion fractures of calcaneal tuberosity. Specifically, our proposal fixes the fracture fragment with a preformed “L-shape” hook plate, using which the short part of the plate can be directly fixed with long screws. Meanwhile, the side of the short plate can also wrap around the fracture, which also plays a key role in fixing the fracture. This study aims to provide an innovative and clinically viable internal fixation approach for calcaneal tuberosity avulsion fractures (commonly known as “beak fractures”), for which we retrospectively analyzed 15 cases of beak fracture in order to assess the efficacy of our proposal.

## Materials and methods

### General information

All the experimental protocols were approved by Sichuan Orthopaedic Hospital institutional review board (KY2021-004-01). We reviewed x-rays and clinical data of all patients of calcaneal avulsion fracture referred to our department from January 2015 to February 2020. All methods were performed in accordance with the relevant guidelines and regulations. Written informed consent for the surgical procedure was obtained from all participants and/or their legal guardians. Informed consent for the retrospective component of this study was formally waived by the Institutional Review Board (IRB). The inclusion criteria defining calcaneal avulsion fracture (beak fracture) originate from clinical diagnosis, to which the fracture shall be fixed with L-shape hook plate. Inclusion Criteria: Acute, closed Beavis type II avulsion fracture of the calcaneal tuberosity. Radiographic evidence of significant displacement, defined as proximal displacement of the fragment > 10 mm on a preoperative lateral radiograph. A fracture fragment with a minimum sagittal length of 15 mm on CT imaging, ensuring sufficient bone stock for plate fixation. Fracture line confirmed by CT scan not to involve the posterior facet of the subtalar joint. Absence of severe, irreversible soft-tissue injury (cases with a grade beyond AO/OTA IC-4 were excluded). Exclusion Criteria: Calcaneal body fractures extending into the subtalar joint. Open fractures or pathological fractures. Associated injuries or medical conditions precluding surgical intervention (e.g., severe peripheral vascular disease).

All relevant clinical data were recorded, including patient age, gender, body mass index (BMI), diabetes status, injury mechanism, details of soft tissue injury, open fracture status, skin condition, preoperative waiting time, operative duration and intraoperative blood loss, time to clinical fracture union, time to independent single-leg heel rise, and follow-up duration. The AO-FAS score, VAS score, and active ankle range of motion were assessed at the final follow-up. Complications were also reviewed, encompassing wound healing issues, infection, reoperation, nonunion, and malunion. To evaluate injury patterns and the severity of displacement, preoperative examinations—including lateral and axial radiographs of the calcaneus, as well as CT scans (axial and three-dimensional reconstructed images)—were performed for all 15 patients.

#### Surgical techniques: internal fixation using preformed “L-shape” hook plate

**Figure 1:**
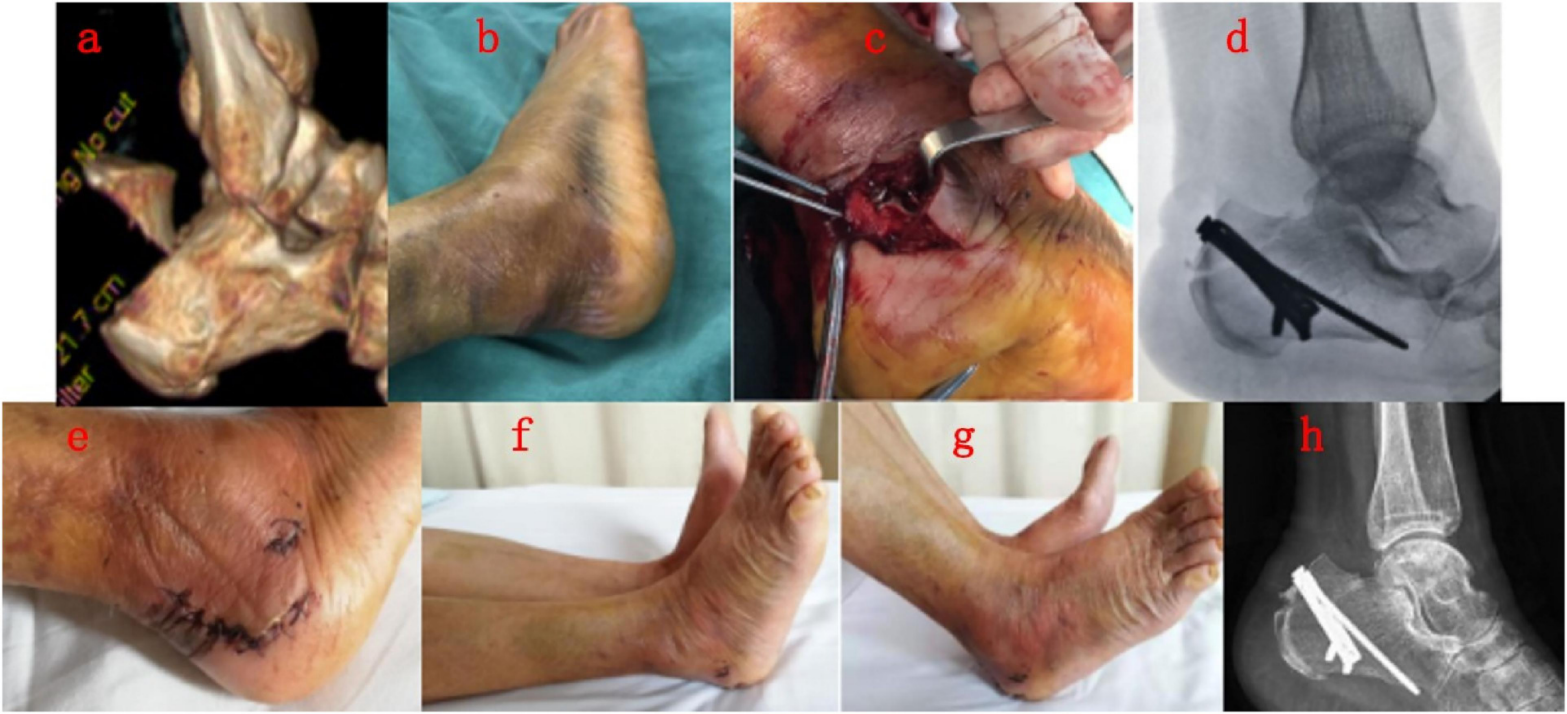
**(a,b)** A 69-year-old female suffered high-altitude falling and resulted in avulsion fracture of calcaneal tuberosity on her right foot; **(c,d)** open reduction and internal fixation with preformed “L-shape” hook plate; **(e)** physical image of surgical incision at discharge; **(f,g)** physical image of ankle dorsiflexion function at discharge; **(h)** healed fracture at 9 weeks postoperatively.

**Figure 2:**
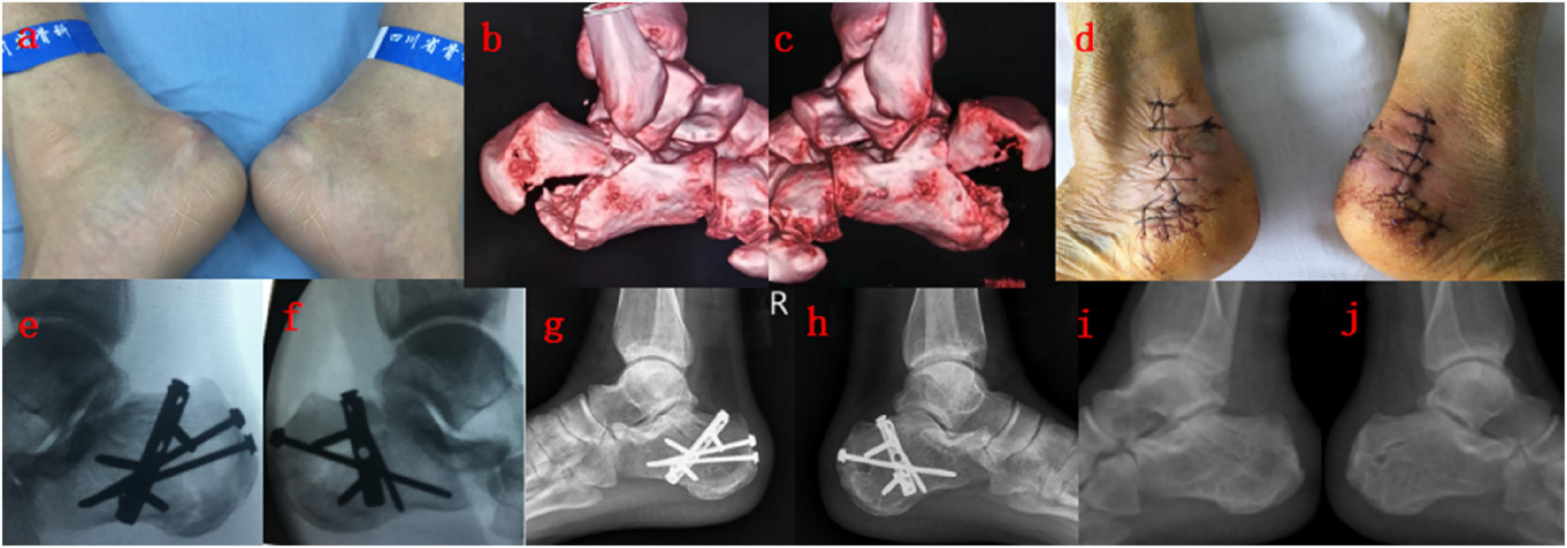
**(a–c)** A 45-year-old man suffered bilateral beak fracture when jumping from high altitude, he underwent emergency surgery, to whom open reduction and internal fixation with “L-shape” plate were conducted; **(d)** incision condition at discharge; **(e,f)** intraoperative radiograph; **(g,h)** x-ray radiograph at 11 months after surgery; **(i,j)** after removing the internal fixation.

**Figure 3:**
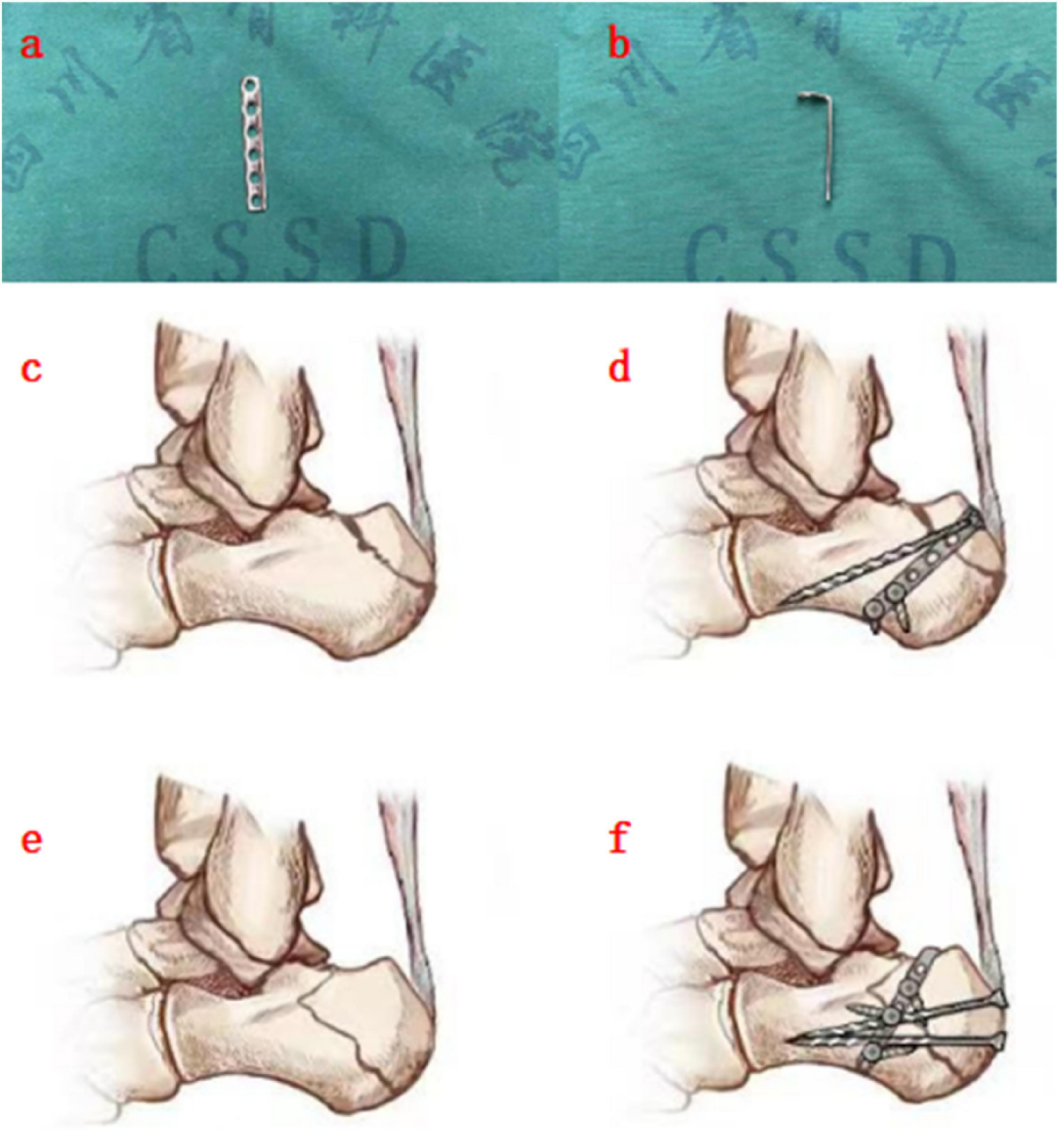
Schematic diagrams demonstrating the fixation of two different subtypes. **(a,b)** 1/4 stainless steel tubular plate with preformed screw holes; **(c)** avulsion fracture of calcaneal tuberosity (beak fracture) with relatively smaller fragment; **(d)** the plate is placed on the top gap of the insertion of Achilles tendon to resist traction forces of the Achilles tendon; **(e)** avulsion fracture of calcaneal tuberosity (beak fracture) with relatively larger fragment; **(f)** the plate is placed on top of the fracture fragment, to which 1–2 screws are attached to fix the fracture below the Achilles tendon insertion for strengthening the fragment fixation.

After admission, patients with closed calcaneal beak fractures finished necessary examinations to exclude surgical contraindications, after which they underwent emergency surgery. During the surgery, general anesthetic and nerve blocking anesthesia were adopted altogether, the patient was placed in lateral decubitus position, to whom tourniquets and sterilized surgical drapes were applied. A modified short “L-shape” incision was performed on the lateral side of the calcaneus, and the subcutaneous tissue of the skin was opened. A lateral approach was conducted with respect to the calcaneus, accompanying with elevation of full-thickness skin flaps, after which the fracture was identified and cleared. Subsequently, one assistant placed the ankle joint in plantar flexion, and the surgeon used two point-shaped reduction forceps to reduce the displaced “beak fragments”. After satisfactory reduction of the fracture and temporary cross-fixation with Kirschner wires, fragments were covered with a 1/4 tubular plate of stainless steel which is preformed with “L-shape”. Then from the screw holes perpendicular to the fracture line on the hook plate, 3.5 mm screws were implanted to the plantar side of the calcaneus, and the fracture fragments were fixed with compression. After that, eccentric plate fixation was conducted by placing screws through screw holes on the lateral side of the calcaneal body, the screws at the hook of the plate were tightened again, and then the Kirschner wires were pulled out. Intraoperatively, the ankle was passively moved to test the stability of the fracture fixation, after which the skin was sutured and drainage strip was placed. After the operation, the ankle joint was bandaged in the functional position for protection and recovery (Figures 1–3).

### Postoperative management

Postoperatively, the ankle was maintained in neutral position with below-the-knee cast for the first 4 weeks. Active ankle exercises were encouraged after removing the plaster. Heel-rise weight-bearing exercises of the ankle can be started after 6 weeks of surgery. Nevertheless, the principle of weight-bearing without pain must be followed first. x-ray imaging with lateral and axial view were performed to examine the fracture healing status at first month, second month and third month respectively after the operation. Full weight-bearing walking without padded shoes can be allowed if clinical fracture healing criteria are achieved.

### Function outcome assessment

Functional recovery was evaluated using the American Orthopaedic Foot & Ankle Society Ankle-Hindfoot Scale (AOFAS-AH) and the Visual Analog Scale (VAS) for pain. These scores were recorded preoperatively and at the final follow-up.

Radiographic Evaluation: Standard lateral and axial radiographs of the calcaneus were obtained at each visit to assess for loss of reduction, implant failure (screw loosening/breakage, plate deformation), and fracture healing progression.

Clinical Examination: Performed by the attending surgeon to evaluate wound healing (dehiscence, necrosis, infection), neurological status (sural nerve sensation), and signs of deep vein thrombosis.

Adjunct Diagnostic Tests: Laboratory tests (complete blood count, C-reactive protein) or Doppler ultrasound were performed if clinical suspicion of infection or deep vein thrombosis arose, respectively.

Patient-Reported Outcomes: Direct questioning regarding any new or persistent symptoms.

### Definition and assessment of fracture union

Fracture union was determined using a composite definition incorporating both clinical and radiographic criteria, as follows: Clinical Criteria for Union (all must be met):Absence of localized tenderness or pain on axial percussion at the fracture site. No abnormal mobility upon manual stress testing of the non-articular fracture segment. Functional recovery, specifically: the ability to walk with full weight-bearing without pain, and to perform a painless single-leg heel raise. Maintenance of the above conditions for a consecutive two-week observation period (calculated retrospectively from the first day these criteria were met).Radiographic Criteria for Union (either criterion accepted): Blurring or disappearance of the fracture line on standard lateral and axial radiographs of the calcaneus. Presence of continuous bridging callus across the fracture line on both radiographic views. Operational Definition for This Study: In this study, a fracture was considered to have achieved “clinical healing” at the time point when it satisfied Clinical Criteria 1, 2, and 3 above, and concurrently met at least one of the Radiographic Criteria. The healing time for each patient was confirmed by consensus of two independent observers (the attending surgeon and a senior resident) applying this defined protocol (14).

### Statistical method

SPSS 20.0 software package was used for statistical analysis, and the measurement data were expressed using mean ± standard deviation. The paired t-test was adopted to compare the preoperative and postoperative samples. The significance level was set to *P* < 0.05.

## Results

This study included a total of 15 patients with calcaneal tuberosity avulsion fractures (beak fractures) who were treated using our designed L-shaped hook plate technique. The mean follow-up duration was 17.1 ± 6.0 months (range: 12–36 months). Among all patients, there were 6 females and 9 males, with an age range of 29–69 years (mean age: 52.9 ± 11.2 years). The mean BMI of the enrolled patients was 24.3 ± 2.2 (range: 19.8–28.1). Fractures occurred in the right lower limb in 9 cases, the left lower limb in 5 cases, and 1 rare case presented with bilateral fractures. Among these 15 calcaneal fractures, the primary injury mechanism was falls from height (9 cases), followed by slips and falls on stairs (5 cases), and 1 case resulted from accidental injury while crossing a stream. Of all cases, 11 patients underwent emergency surgery, while the remaining 4 patients, due to concurrent medical conditions requiring evaluation and absence of severe skin irritation upon admission, received elective surgery 2–4 days after admission. The mean time from outpatient visit to surgery was 16.1 ± 10.8 h (range: 8–42 h). The mean operative duration was 58.7 ± 20.7 min (range: 40–130 min), and the mean intraoperative blood loss was 26 ± 9.1 mL (range: 10–50 mL).

Postoperatively, none of the 15 patients exhibited the following complications: poor wound healing, flap necrosis, wound dehiscence, hematoma or infection, plate exposure, sural nerve injury, venous thromboembolic events, loss of reduction, or implant failure. In this study, all 15 cases achieved clinical union within 8–13 weeks postoperatively (10.5 ± 1.4 weeks). The patients regained the ability to perform a single-leg heel raise on the affected side at 3.7 ± 0.7 months after surgery. Regarding postoperative functional assessment, the preoperative VAS score and AOFAS-AH score were 5.7 ± 0.6 and 24.0 ± 9.9, respectively, while at the final follow-up, the postoperative VAS and AOFAS-AH scores improved to 1.3 ± 0.5 and 93.8 ± 5.2, respectively (*p* < 0.001). Furthermore, all patients reported an ankle range of motion of 57.8 ± 7.1°, which was comparable to the unaffected/contralateral side (59.5 ± 7.3°), with no subjective complaints of impaired recovery. All 15 patients were able to wear regular shoes without significant pain or discomfort and could engage in normal daily activities (Table 1).

**Table 1:** Comparison of preoperative and final follow-up functional outcomes.

Outcome measure	Preoperative score (mean ± SD)	Final follow-up score (mean ± SD)	Mean improvement (95% CI)	*P* Value
AOFAS-AH score	24.0 ± 9.9	93.8 ± 5.2	69.8 (64.2–75.4)	<0.001
VAS pain score	5.7 ± 0.6	1.3 ± 0.5	−4.4 (−4.7 to −4.1)	<0.001

## Discussion

### Performing emergency surgery as early as possible is a major way to avoid soft tissue necrosis and/or improve soft tissue condition

Avulsion fractures of the calcaneus are primarily classified into three distinct types: the “sleeve” fracture, the conventional “beak” fracture, and the infrabursal fracture located at the middle third of the posterior tuberosity (3, 15). Displaced posterior calcaneal tubercle fracture, especially the abovementioned second type (“beak” fracture), can easily result in local skin and soft tissue pressure necrosis and in ulcers (16). Furthermore, fracture blisters and deep abrasions affect clinical treatment in making decisions, by which the good opportunity of in-time surgery may be delayed. It has been reported that delayed fracture treatment can cause severe skin complications (17, 18). When the fracture displacement exhibits local bony bulge, emergency surgery must be performed as soon as possible. In this study, eleven of the fifteen patients underwent emergency surgery, the interval between injury and emergency surgery in patients who admitted to the hospital was a minimum of 8 h and a maximum of 15 h, with the average time of 9.67 h. All of them were accompanied by closed soft tissue injuries of different degrees. According to AO soft tissue classification, five cases were IC-2, six cases were IC-3, and four cases were IC-4. It must be emphasized that emergency surgery should be performed whenever possible. If immediate surgery is contraindicated due to objective factors (e.g., as in the four IC-2 cases in this series with medical comorbidities necessitating anesthesia risk assessment), priority must be given to applying a plantarflexion cast on the anterior lower leg to avoid soft tissue irritation. In addition, it has been generally acknowledged that if non-surgical treatment for avulsion fracture of calcaneal tuberosity does not achieve its expected clinical effect, an alternative of open reduction and internal fixation is strongly recommended (19, 20). When surgical conditions can be guaranteed, the surgery shall be performed immediately.

### Strong fixation reduces recovery time while preventing internal fixation from failure

There are various studies on choosing the optimal internal fixation for performing surgery (21, 22). Avulsion fracture of calcaneal tuberosity has been treated with diverse approaches such as screw fixation, suture anchor fixation, and tension band wiring (23). Squires et al. recommended the use of steel wire and tension bands for fixation (24). Yoshida et al. reported that lag screws can be adopted to fix calcaneal tuberosity avulsion fracture (25). However, complications were often reported, which included avulsed fragment displacement, wound dehiscence, and skin irritation. With respect to the patients with small fractures, they were treated with screw fixation. Unfortunately, the article reported one case of failure of the internal fixation using screws (26). As for the patients with large fragments, particularly for those with the fracture line extending to subtalar joint, some researchers adopted conventional lateral calcaneal plate with larger incision (26). The utilization of cannulated cancellous screws and titanium wire not only provided strong internal fixation without any skin or incision problems that may be caused by previous methodologies, but enabled earlier postoperative recovery with range of motion and weight-bearing exercises (27). However, the above approach conducted a posterior Achilles tendon vertical midline curved incision and a small lateral plantar incision, which undoubtedly increased the wound area. In order to treat a case of chronic “beak fracture”, Laxman Rijal performed Z-plasty of Achilles tendon, through which the proximal stump of Achilles tendon was reinforced by non-absorbable polyester suture with a 4.5 mm cancellous screw placed transversely in calcaneus (28). Specifically, the reinforcement sutures were positioned to secure the tendon lengthening. Fragments were fixed with 4.5-mm cancellous cannulated screws and washers. Before allowing the patient to gradually begin weight bearing, the reinforcement sutures were percutaneously removed under local anesthesia on an outpatient basis (28). Ramanujam CL reported that through using Steinmann pins and Ilizarov external fixation, significant traumatic calcaneal avulsion fractures with concomitant Achilles tendon ruptures can be successfully reduced (29). Nevertheless, it must be noted that this technique still has one disadvantageous effect of cutting a second plantar incision, which may potentially cause skin necrosis or irritation (29). Agni et al. applied locking compression hook plate (LCP) to treat avulsion fracture of calcaneal tuberosity (30). They further discovered that this approach can also offer stable fixation, however, the LCP hook plate is comparatively expensive. Ernst et al. adopted “hurricane strap” technique (An A.L.P.S dorsal midfoot fusion plate is bent into an L shape with a traditional longer “L” incision for fixation) to fix tongue-type calcaneal fracture (31). Ding et al. introduced a new technique for successfully treating calcaneal tuberosity fracture using a fixation of 180-degree microplate (5). However, this research paper is only a case report and provides with limited theoretical foundation.

In contrast to the abovementioned treatment and fixation, the incision involved in our proposal is not performed at the heel or on the sole of the foot. Instead, we choose an improved and shorter “L-shape” incision on the median line of posterior edge of Achilles tendon and fibula. The incision is kept a certain distance away from the injured skin surface to prevent the soft tissue from being irritated by the displaced fracture fragment at the heel. Through performing the above approach, no necrosis of skin or soft tissues are observed in our results. Specifically, our proposed method adopts a one-fourth tubular plate, which is not so expensive compared with locking plates. During the operation, the plate is preformed to customize it hook-shaped, thereby increasing the holding force of the plate on the fragment. One to two screws can be mounted into the hook at the fragment for compression fixation. Apart from the pressure generated by the screws, the plate can offer pressure on the entire surface of the fracture through the hook. In addition, the eccentric screw can be inserted in the lateral wall of the calcaneus to make the hook fit better with the fracture. Inserting several screws in the lateral wall of the calcaneus better fixes the plate while increasing the stability of the fracture fragment, thereby avoiding internal fixation failure. Lots of patients involved in this study have realized early postoperative functional exercises.

### Study limitations

Our findings should be interpreted in light of several limitations. The study is retrospective in design and involves a relatively small cohort of 15 patients, which may affect the statistical strength and broader applicability of the conclusions. Moreover, the lack of a control group treated with other fixation methods limits our ability to make comparative claims regarding the efficacy of the L-shaped hook plate. All surgeries were performed by a single surgical team at one institution, raising the possibility of technique-related bias and potentially restricting the generalizability of the results. Although the mean follow-up of 17.1 ± 6.0 months is adequate for assessing early to midterm outcomes, it remains too short to evaluate long-term risks such as posttraumatic arthritis or late hardware failure. Lastly, functional assessment relied in part on patient-reported instruments, which are inherently subjective and could introduce assessment bias. Future prospective, multicenter studies with larger sample sizes, control groups, and extended follow-up will be valuable to confirm these preliminary findings and further define the role of this technique in clinical practice.

## Conclusions

Emergency open reduction and internal fixation is recommended for calcaneal avulsion fractures to prevent flap necrosis. For Beavis type II fractures, the preformed L-shaped hook plate demonstrated satisfactory stability and functional recovery in this case series. This technique represents a potential alternative for the surgical management of calcaneal tuberosity avulsion fractures. The preliminary outcomes from these cases support further investigation into its clinical application. However, the absence of a control group limits definitive comparative conclusions. Future studies, including biomechanical evaluations and larger comparative trials, are warranted to more comprehensively assess the efficacy and generalizability of this method.

## Data Availability

The original contributions presented in the study are included in the article/Supplementary Material, further inquiries can be directed to the corresponding author.
